# A Thermal Skin Model for Comparing Contact Skin Temperature Sensors and Assessing Measurement Errors

**DOI:** 10.3390/s21144906

**Published:** 2021-07-19

**Authors:** Braid A. MacRae, Christina M. Spengler, Agnes Psikuta, René M. Rossi, Simon Annaheim

**Affiliations:** 1 Empa, Swiss Federal Laboratories for Materials Science and Technology, Laboratory for Biomimetic Membranes and Textiles, 9014 St. Gallen, Switzerland; braid.macrae@rmit.edu.au (B.A.M.); agnes.psikuta@empa.ch (A.P.); rene.rossi@empa.ch (R.M.R.); 2 Exercise Physiology Lab, Department of Health Sciences and Technology, ETH Zurich, 8057 Zurich, Switzerland; christina.spengler@hest.ethz.ch; 3 Centre for Materials Innovation and Future Fashion, School of Fashion and Textiles, RMIT University, Melbourne 3056, Australia; 4 Zurich Center for Integrative Human Physiology (ZIHP), University of Zurich, 8057 Zurich, Switzerland

**Keywords:** human skin temperature, thermometry, temperature sensor, measurement bias, measurement error, skin model, thermal strain

## Abstract

To improve the measurement and subsequent use of human skin temperature (*T*_sk_) data, there is a need for practical methods to compare *T*_sk_ sensors and to quantify and better understand measurement error. We sought to develop, evaluate, and utilize a skin model with skin-like thermal properties as a tool for benchtop *T*_sk_ sensor comparisons and assessments of local temperature disturbance and sensor bias over a range of surface temperatures. Inter-sensor comparisons performed on the model were compared to measurements performed in vivo, where 14 adult males completed an experimental session involving rest and cycling exercise. Three types of *T*_sk_ sensors (two of them commercially available and one custom made) were investigated. Skin-model-derived inter-sensor differences were similar (within ±0.4 °C) to the human trial when comparing the two commercial *T*_sk_ sensors, but not for the custom *T*_sk_ sensor. Using the skin model, all surface *T*_sk_ sensors caused a local temperature disturbance with the magnitude and direction dependent upon the sensor and attachment and linearly related to the surface-to-environment temperature gradient. Likewise, surface *T*_sk_ sensors also showed bias from both the underlying disturbed surface temperature and that same surface in its otherwise undisturbed state. This work supports the development and use of increasingly realistic benchtop skin models for practical *T*_sk_ sensor comparisons and for identifying potential measurement errors, both of which are important for future *T*_sk_ sensor design, characterization, correction, and end use.

## 1. Introduction

There is enduring and wide-ranging interest in human skin temperature (*T*_sk_), spanning classic physiology research [1,2], general assessment of thermal strain [3], and emerging health monitoring applications [4,5]. Reasons for such interest include the importance of the skin in maintaining thermal homeostasis [6] and the way in which the skin—as the body’s external interface—offers potential insight into other physiological or psychophysical responses or parameters (e.g., [7,8]). While the skin is typically an easily accessible site for measurement, this ease of measurement may belie certain challenges involved in accurately quantifying human *T*_sk_. Indeed, the method used to measure *T*_sk_ can influence the measured outcome [9,10]. With increasing interest in wearable technologies (coupled with the ease of adding temperature sensors to multi-parameter measurement systems) and the associated potential for large and personalized datasets, understanding limitations of measurement techniques is necessary to maximize the validity of data use.

Challenges involved in the accurate measurement of human *T*_sk_ were the focus of reasonable attention during the first half of the 20th century, with bare-wire thermocouples and radiometers commonly constructed and studied (e.g., [11,12,13]). From this early body of work, indications were that, at least under resting conditions, carefully designed radiometers (non-contact method) provided the best estimate of the ‘true’ *T*_sk_ [11,14,15]. Bare-wire thermocouples, consisting of fine wires with diameters less than 0.6 mm, in direct contact with the skin compared well with radiometers (often within ±0.5 °C of the radiometer), with the magnitude of the difference in each case better or worse depending upon variables such as the thermocouple diameter, coverage or method of attachment, applied pressure, and environmental conditions [16,17]. A relatively detailed understanding was developed for the sensors and measurement purposes of the time. However, these delicate thermocouples were, like the radiometer, not practical for meeting general modern demands for measuring human *T*_sk_, particularly during physical activity or under clothing and other equipment or body coverings.

There has been greater availability of commercially made, more convenient, and more robust contact temperature sensors over the late 20th and early 21st centuries. Practical advantages of being able to affix such sensors directly to the skin surface likely contributed to the widespread adoption of the contact method of measurement. However, measurement limitations of these contemporary (and comparatively bulkier) *T*_sk_ sensors are incompletely characterized. Interrelated challenges include that the measurement system may modify the local *T*_sk_ via effects on heat and mass transfer [18] and that the sensor is in contact with other mediums, such as the ambient environment and attachments (e.g., tape), in addition to the skin itself [19,20,21,22]. The interplay between the physical properties of a sensor system, the skin, and the measurement conditions may contribute to the mixed agreement between sensor systems reported in more recent studies [21,23,24,25,26]. Local modifications of human *T*_sk_ have been demonstrated by obstructing evaporative heat transfer (in vivo [27,28]) and by insulating the *T*_sk_ sensor (numerical simulation [18]). However, a detailed practical assessment of both systematic error (bias) and local *T*_sk_ modification under the same measurement conditions is lacking. To this end, laboratory tools that facilitate investigation of measurement error under conditions that approximate end use are warranted, given the impracticality and logistical constraints of using human skin in vivo. Similarly, laboratory tools that facilitate relatively easy and ‘standardized’ intra- or inter-sensor comparisons are required, given the proliferation of monitoring technologies and associated sensor designs and the emerging need for investigating or tracking relative measurement performance within or among those sensors. Laboratory models with metallic surfaces have been used in the past [21,22,29,30,31], typically to capitalize on heat transfer within the material itself, although models that more realistically reflect the thermal properties of human skin would be advantageous.

Here, we sought to develop, evaluate, and utilize a synthetic skin model with realistic thermal properties as a tool for benchtop *T*_sk_ sensor comparisons and assessments of measurement error. Following production of the thermal skin model, our aim was to (1) evaluate the suitability of this skin model as a practical substitute to human skin for inter-sensor comparisons of contact *T*_sk_ sensors. This evaluation was performed by using three types of contact *T*_sk_ sensors and comparing model-derived inter-sensor differences to the corresponding inter-sensor differences determined on human skin in vivo during rest and cycling exercise. Next, utilizing small reference temperature sensors embedded within the skin model, we sought to better understand potential measurement errors and other local temperature effects of using contact *T*_sk_ sensors. Thus, the interrelated second and third aims were to (2) characterize the magnitude of local temperature disturbances at the model surface caused by the presence of selected *T*_sk_ sensors and an attachment tape and (3) characterize the magnitude of measurement bias when using selected *T*_sk_ sensors.

## 2. Materials and Methods

### 2.1. Orientation

Following the conceptualization and production of a thermal skin model, this model was used as a platform for detailed *T*_sk_ sensor assessment. Model experiments involved two parts: (1) inter-sensor comparisons and (2) characterization of local temperature disturbances sensor bias. A human trial was undertaken for in vivo inter-sensor *T*_sk_ comparisons, with the outcome compared to the model inter-sensor comparisons. Because specific outcomes from the human trial were used to inform part of the data analysis in the skin model component, the human trial (Section 2.4) is described below before the skin model experiments (Section 2.5).

### 2.2. Production of the Skin Model

A thermal skin model (outer dimensions 300 mm × 300 mm × 10 mm) was made using a dark gray silicone elastomer (polydimethylsiloxane; Sylgard 170; Dow Corning, Midland, MI, USA). Selected properties of this skin simulant were (reported in more detail by Zhai et al. [32]): thermal conductivity ~0.23 W/(m∙K), thermal diffusivity ~12.8 × 10^−8^ m^2^·s^−1^, emissivity ~0.9, and density ~1350 kg·m^−3^, which compare quite well with human epidermis: 0.21–0.63 W/(m∙K), 4.9–14.6 × 10^−8^ m^2^·s^−1^, 0.96–0.99, and ~1200 kg·m^−3^ for the same properties, respectively.

**Figure 1:**
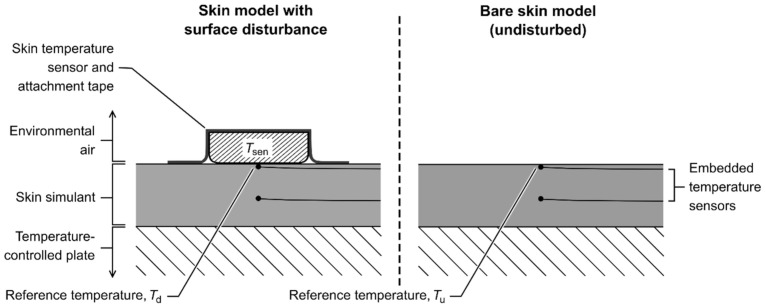
Cross-section of the skin model at the measurement site, showing the surface position of the skin temperature sensor and/or tape when used (left) and the same section of the model in the state ‘unmeasured’ by the skin temperature sensor (right). The top embedded temperature sensor (directly subsurface) was used as the reference surface temperature, becoming the *disturbed* reference temperature (*T*_d_) when a surface skin temperature sensor was present (left) and the *undisturbed* reference temperature (*T*_u_) when the model surface was bare (right). This setup enabled characterization of the relationships among the temperature of the surface skin temperature sensor (*T*_sen_), *T*_d_, and the corresponding *T*_u_ under otherwise equivalent conditions.

The silicone elastomer was prepared from the precursors (Sylgard 170A/170B) at a ratio of 1:1 by mass, mixed, then de-gassed in a vacuum chamber at ambient lab temperature; the mix was carefully poured into a mold and cured under ambient lab conditions (22.8 ± 0.6 °C, 63 ± 3% relative humidity; RH). Small glass bead thermistors (bead diameter 0.8 mm; type B57540G1, 10 kΩ; Epcos AG, Munich, Germany) were embedded in the skin simulant, with the primary sensor—that was used as the surrogate of the model surface temperature (reference temperature; *T*_ref_)—being directly subsurface (within 1 mm of the surface). Being directly subsurface facilitated the measurement of *T*_ref_ with coverage by a surface *T*_sk_ sensor and/or attachment (disturbed *T*_ref_ (*T*_d_)) and without any coverage (undisturbed *T*_ref_ (*T*_u_)). A schematic is given in Figure 1, and more detail about the model setup is provided in Appendix A, including the selection of *T*_u_. Thermistors were used in the skin model because of their high sensitivity to changes in temperature within the narrow temperature range physiologically relevant for humans.

The thermistors embedded in the skin simulant were calibrated in situ using a calibration chamber (OptiCal; Michell Instruments Ltd., Cambridge, UK) and an externally calibrated precision laboratory Pt100 sensor and thermometer as the reference measurement (Kelvimat 4323; Burster GmbH, Gernsbach, Germany; resolution 0.01 °C, uncertainty 0.02 °C; recorded at 5 s intervals). Data from the embedded thermistors were recorded at 10 s intervals with a resolution of ≤0.01 °C using a data logger (Squirrel SQ2020-2F8; Grant Instruments Ltd., Cambridge, UK). The calibration procedure covered the temperature range of 15–40 °C with stepped-intervals of ~5 °C and ≥80 min duration per step. Eight-minute steady states were identified where the steady state standard deviation (SD) was <0.007 °C for the reference, and mean data over these eight-minute periods were used to generate sensor-specific corrections using linear least-squares regression (Appendix B).

Temperature control of the skin model was achieved using an aluminum plate immediately below the skin simulant; the aluminum plate had internal channels, which were perfused using a 25 L water bath and circulation thermostat (RM 25 S; Lauda GmbH & Co. KG, Lauda-Königshofen, Germany). The setup was capable of a steady state stability of SD <0.01 °C [22].

### 2.3. Skin Temperature Sensors and Attachment Tape

**Figure 2:**
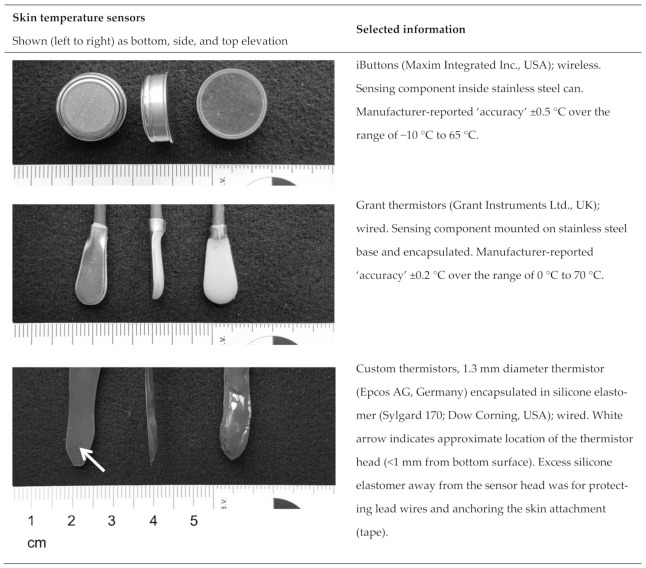
Skin temperature sensors and selected information.

Three types of *T*_sk_ sensors were used throughout this work (Figure 2):

- Maxim iButtons (DS1922L, Maxim Integrated Inc., San Jose, CA, USA). The surface of the iButton with a rounded edge was used as the contact surface [23].
- Grant thermistors (EUS-U-VS5-0, Grant Instruments Ltd., Cambridge, UK).
- Custom thermistors, each consisting of a glass-encapsulated NTC thermistor (type B57550G1, 10 kΩ; diameter 1.3 mm; Epcos AG, Germany) set inside a small volume of silicone elastomer encapsulant (Sylgard 170; Dow Corning, Midland, MI, USA).

The iButtons and Grant thermistors were selected based on being two of the most common commercial sensors used for measuring human *T*_sk_ in published studies involving exercise and other physical activity over the period 2011–2016 [10]. The custom thermistors were used as a model for investigating the idea of having thermal properties of the *T*_sk_ sensor matching the medium it is measuring (the silicone elastomer used to encapsulate the thermistor in the custom sensor was the same silicone elastomer used to make the skin simulant; Section 2.2).

All data from *T*_sk_ sensors were recorded at 10 s intervals for sensor calibration and during experimental sessions. Data from the custom thermistors and Grant thermistors were collected with a resolution of ≤0.01 °C using a data logger (Squirrel SQ2020-2F8). Data from the iButtons were self-logged with a resolution of 0.0625 °C. *T*_sk_ sensors were calibrated prior to each set of experimental sessions (skin model and human trial) using the calibration chamber, Pt100 sensor and thermometer, and procedure as described for the thermistors embedded in the skin simulant (Section 2.2 and Appendix B). During each calibration run, *T*_sk_ sensors were arranged spatially to ensure they avoided contact with each other and the chamber walls.

For both sets of experiments (skin model and human trial), a single layer of porous medical tape was used to attach the *T*_sk_ sensors (Hypafix^®^, 16002). The tape was white, non-woven polyester with acrylic adhesive (thickness, 0.25 ± 0.03 mm; mass-per-unit area, 76 ± 1 g·m^−2^; BSN Medical GmbH, Hamburg, Germany). The tape size used was 35 mm × 50 mm for the iButton and 30 mm × 50 mm for the Grant and custom thermistors, keeping the total surface area of the measured surface (skin or model) covered approximately the same irrespective of the sensor used.

### 2.4. Human Trial—In Vivo Skin Temperature Sensor Comparison

#### 2.4.1. Participants

Fourteen male adults participated in the human trial (mean ± SD; age, 28.1 ± 3.4 y; mass, 79.0 ± 5.1 kg; standing height, 1.84 ± 0.05 m). All participants were non-smokers, apparently healthy, and exercised habitually (typically ≥3 sessions/week exceeding 45 min/session). Written informed consent was obtained from all participants. The ethics committee of Eastern Switzerland (Project ID: 2017-01376, EKOS 17/129) approved the study, and all procedures performed were in accordance with the ethics committee and with the 1964 Declaration of Helsinki and its later amendments.

#### 2.4.2. Procedures

**Figure 3:**
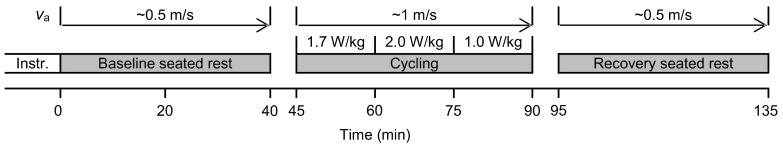
Schematic of the human trial experimental protocol. Minutes 40–45 and 90–95 were used for transitioning between the chair and cycle ergometer and to check for sensor or attachment issues. Intsr., instrumentation; *v*_a_, air velocity.

There was one experimental session per participant (Figure 3). Participants were requested to abstain from alcohol and caffeine within 12 h of the trial, strenuous exercise within 48 h of the trial, and to have a light meal ~1 h before reporting to the laboratory at 0800 (n = 11) or 1400 h (n = 3). Experiments were completed in an environmental chamber for controlled environmental conditions (temperate conditions, targeting 24 °C and 50% RH; actual measured conditions across experimental sessions were 23.6 ± 0.3 °C, 46 ± 3% RH). Air velocity of ~0.5 m·s^−1^ during seated rest and ~1 m·s^−1^ during exercise was provided by two vertically aligned 0.25 m diameter fans, with those fans 2.3 m and 1.65 m in front of the participant during rest and exercise, respectively. Rest comprised sitting on an office chair, and exercise consisted of cycling on a stationary ergometer (cyclus 2; RBM elektronik-automation GmbH, Leipzig, Germany). Exercise was moderate intensity for the first 30 min (15 min at fixed-load of 1.7 W·kg^−1^ body mass (FL1), 15 min at 2.0 W·kg^−1^ (FL2)) followed by a 15 min light-intensity warm down (1.0 W·kg^−1^; FL3). Pedaling cadence was 75 ± 3 rpm throughout.

Participants wore their own sport shoes, socks, and running shorts and an experimenter-supplied standardized short-sleeve 100% cotton t-shirt (olive green) in the appropriate generic size (small, medium, or large; garment ease, and therefore fit, was not otherwise characterized). The t-shirt fabric was conditioned and characterized at 20 ± 2 °C and 65 ± 4% RH [33] in accordance with the corresponding standard test method: thickness [34], 0.83 ± 0.02 mm; mass per unit area [35], 189 ± 4 g·m^−2^; air permeability [36], 733 ± 19 L/(m^2^·s); and thermal resistance [37], 0.030 ± 0.001 m^2^·K/W.

#### 2.4.3. Experimental Measurements

Participants were instrumented in the test environment. The *T*_sk_ (three sensor types; Figure 2), and RH at the skin surface were each measured on the left side of the body at four sites [38]: upper chest (pectoralis major), lateral arm (at widest circumference), anterior thigh (midway along the femur), and anterior leg (muscle belly of tibialis anterior). RH at the skin surface, and the corresponding temperature (for subsequent calculation of absolute vapor pressure), were measured at 10 s intervals using an integrated hygrometer and thermistor (SHT15 sensor and MSR12 logger; MSR GmbH, Seuzach, Switzerland), which had been calibrated over the range of 40–87.5% RH and 15–40 °C (OptiCal calibration chamber) within two months of commencing the study.

The measurement sites were cleaned with an isopropyl swab and lightly shaven ~30 min before instrumentation. For the relative position of the three *T*_sk_ sensors at each body site, a 50 mm × 50 mm square was marked on the skin then separated into four equal quadrants, 25 mm × 25 mm each, with each sensor then randomly allocated to a quadrant [25]. (A sensor used to measure next-to-skin microclimate air temperature was allocated to the remaining quadrant; this measurement was practically challenging in the context of body movement, and, due to insufficient confidence in the consistency of the sensor distance from the skin surface, the microclimate temperature data are not reported here.) The RH sensor at each body site was always positioned ~25 mm superior to the square described. The sensors at the chest were covered in all trials, whereas the sensors at the arm, thigh, and leg were always uncovered (the t-shirt left arm had been cropped accordingly). Sensors were affixed to the skin using a single layer of medical tape (described in Section 2.3).

Heart rate was recorded at 5 s intervals (S810i HR monitor; Polar, Kempele, Finland). Near-nude mass (including underwear) was measured immediately before and after entering and exiting the environmental chamber (ID5 Multi range; Mettler Toledo, Columbus, OH, Switzerland).

#### 2.4.4. Calculations

Weighted-mean *T*_sk_ (°C) was calculated for each *T*_sk_ sensor type using the four measurement sites (Section 2.4.3) as [38]:

Weighted-mean *T*_sk_ = 0.3·*T*_chest_ + 0.3·*T*_arm_ + 0.2·*T*_thigh_ + 0.2·*T*_leg_ (1)

Period means (3 min) were then used for subsequent sensor comparisons: end of baseline rest (mins 36–39 from start of trial), end of each block of fixed-load cycling (mins 57–60, 72–75, and 87–90), and midway through and end of recovery rest (mins 112–115 and 131–134). The 3 min period immediately preceding the onset of sweating was also used, with the timing being specific for each participant. The within-participant, inter-sensor differences were calculated (Grant–custom; iButton–custom; iButton–Grant) and summarized as group-mean differences with corresponding 95% confidence intervals (CI). CI were calculated using the standard error of the difference and the corresponding critical value from the *t*-distribution; CI were unadjusted [39].

RH and corresponding temperature data were used to calculate absolute vapor pressure (kPa) in accordance with formulae given elsewhere [40]. The onset of sweating was estimated by identifying a distinct increase in the absolute vapor pressure (>0.5 kPa) after the beginning of exercise [28]. ‘Continuous’ data (10 s intervals) of absolute vapor pressure from the four body sites were assessed, and the earliest of the four sites was taken as the onset of sweating.

### 2.5. Thermal Skin Model Experiments

#### 2.5.1. Procedures

Experiments were performed in an environmental chamber at 23.8 ± 0.1 °C and 42 ± 2% RH throughout (radiant temperature ≅ air temperature), closely matching the environmental temperature and humidity used for the human trial (means within 0.2 °C and 4% RH). There were two air velocity conditions: ~0.5 m·s^−1^ (with enclosure and fans) and ~0.2 m·s^−1^ (ambient air movement inside the environmental chamber; Appendix A).

An experimental run consisted of measurements during stepwise increases in the temperature of the skin model (five temperature steps at 30 min per step). The steps were set up such that the surface temperature of the skin model began at approximately the same temperature as the environmental air temperature (i.e., ~0 °C gradient from surface to environment) and finished at ~40 °C (0.5 m·s^−1^ condition; temperature gradient range of ~0–16 °C) or ~42 °C (0.2 m·s^−1^ condition; temperature gradient range of ~0–18 °C); the difference in the temperature range between air velocity conditions was due to the greater heat flux through the skin model with the higher air velocity for a given supply temperature of the underlying aluminum plate.

There were six surface-coverage conditions, corresponding to the sensor and/or attachment configuration used at the model surface directly above the model *T*_ref_ sensor: (1) custom thermistor and tape, (2) Grant thermistor and tape, (3) iButton and tape, (4) iButton only (i.e., without fixation), (5) tape only, and (6) no covering (bare model surface). The tape used to attach the *T*_sk_ sensors was as described in Section 2.3; for the ‘tape only’ condition, the tape dimensions were 25 mm × 50 mm to keep consistent the area of the skin model surface covered. The iButton was used to investigate the effect of the attachment tape (sensor covered with tape versus sensor without tape coverage), because this sensor, being wireless, was able to rest on the model surface under its own weight. The bare surface condition, with no *T*_sk_ sensor or tape, was used to establish the reference *T*_u_ (see Appendix A.2 for more detail).

Each of the six surface-coverage conditions (with n = 5 distinct replicates for each) was repeated under each air velocity condition, giving 60 (6 × 5 × 2) experimental runs. Testing was performed in blocks split by air velocity condition, with the sequence within each block ordered by replicate and randomized by coverage condition (i.e., all coverage conditions for replicate 1 were completed before moving on to replicate 2, and so on).

#### 2.5.2. Calculation of Steady State Data

Steady state means for temperature data were calculated over minutes 21–29 (of 30) within each step of each run, giving paired *X*–*Y* data (*T*_sk_ sensor and *T*_ref_, respectively) for runs in which *T*_sk_ sensors were also used. All steady states were confirmed by a within-sensor SD of <0.05 °C. The *T*_ref_ data (*T*_d_ and *T*_u_) were then corrected to account for the *T*_ref_ sensor being subsurface (within the first 1 mm of the skin model), rather than at the surface, itself. Assuming an approximate linear temperature gradient, these location errors were estimated for each steady state in each individual run using Fourier’s Law and the corresponding data from the embedded sensor at a second depth (Figure 1). The magnitudes of these estimated location errors over all experimental runs were 0.00–0.39 °C. (The *T*_sk_ sensor data were *not* corrected in this way, consistent with normal use.)

#### 2.5.3. Comparison of Thermal Skin Model with Human Trial

The suitability of the skin model was explored by comparing the model-derived mean differences among the three *T*_sk_ sensors to those obtained in vivo from the human trial. Because the absolute temperature of the surface being measured (i.e., skin model or human skin) would itself influence the magnitude of the respective inter-sensor differences, inter-sensor comparisons from the skin model needed to be calculated at surface temperatures that corresponded to the surface (i.e., skin) temperatures in the human trial. Therefore, linear regression was used to estimate the *T*_sk_ sensor values for a model surface temperature that approximately matched each period mean *T*_sk_ from the human trial (an example to supplement the following description is given in Appendix C). 

The relationship between each individual *T*_sk_ sensor and the model reference temperature under each air velocity condition were first determined using simple linear regression (ordinary least squares) as:

*Y*′ = *b*_0_ + *b*_1_·*X* (2)

where *Y*′ is the estimate of model reference temperature (*T*_u_; °C), *b*_0_ is the intercept (°C), *b*_1_ is the slope coefficient, and *X* is the *T*_sk_ sensor temperature (°C). All *X*–*Y* data were suitable for linear characterization: for each equation produced, the coefficient of determination (*R*^2^) was >0.99, and the typical error of the estimates (mean residual error from the fitted regression line) was <0.01–0.05 °C. 

The custom thermistors were used to establish the model-equivalent temperature approximating each period mean from the human trial (Section 2.4.4), because these were the *T*_sk_ sensors found to have the least bias (versus *T*_u_) of the three *T*_sk_ sensor types during measurements of the skin model. Accordingly, Equation (2) was used to determine seven model *T*_u_ estimates (*Y*′_1–7_) approximating each of the seven human trial data summary periods, using the custom thermistor group-mean temperatures from the human trial. Next, Equation (2) was rearranged as:

*X*′ = (*Y*′ − *b*_0_)/*b*_1_ (3)

where *X*′ is the *T*_sk_ sensor temperature estimate (°C). For each individual *T*_sk_ sensor within each *T*_sk_ type, and for each air velocity condition, *X*′ was calculated at each of the seven model *T*_u_ estimates corresponding to the human trial (*Y*′_1–7_ from above). This process gave temperature estimates for the three *T*_sk_ sensor types for ‘fixed’ model surface temperatures approximating the human trial. Differences (n = 5) between the *T*_sk_ sensor types were then calculated in the same way as for the human trial (Grant–custom; iButton–custom; iButton–Grant) and summarized as group-mean differences with corresponding 95% CI.

#### 2.5.4. Local Temperature Disturbance and Sensor Bias

The disturbance of the local skin model temperature (°C) caused by the presence of the surface *T*_sk_ sensor and/or attachment was calculated for each steady state within each experimental run (excluding bare surface runs) as:

disturbance = *T*_d_ − *T*_u_ (4)

where *T*_d_ is the model reference surface temperature when disturbed by the surface *T*_sk_ sensor and/or tape attachment (°C), and *T*_u_ is the model reference surface temperature for the corresponding steady state in an undisturbed state (°C).

The measurement bias of each surface *T*_sk_ sensor was considered here in two forms: difference from the underlying disturbed reference surface temperature (bias from *T*_d_) and difference from the corresponding undisturbed reference temperature (bias from *T*_u_). For each *T*_sk_ sensor, at each steady state within each applicable experimental run, bias was calculated as:

bias = *T*_sen_ − *T*_ref_ (5)

where bias (°C) is either bias from *T*_u_ or bias from *T*_d_ (depending on the *T*_ref_ used), *T*_sen_ is the temperature of the surface *T*_sk_ sensor (°C), and *T*_ref_ is either *T*_d_ or *T*_u_ (°C) from the same or corresponding steady state, respectively. 

Estimates of local temperature disturbance and sensor bias were summarized as mean and 95% CI.

## 3. Results

### 3.1. Human Trial

#### 3.1.1. Heart Rate and Onset of Sweating

All participants completed the experimental session. The cycling intensity was sufficient to provide moderate cardiovascular strain: heart rate increased from 70 ± 11 beats·min^−1^ at the end of baseline rest to 129 ± 14 and 145 ± 15 beats·min^−1^ at the end of the fixed-load intensities 1 and 2, respectively, then reduced to 121 ± 14, 82 ± 15, and 79 ± 11 beats·min^−1^ at the end of the warm-down cycling, middle of recovery rest, and end of recovery rest, respectively. The estimated onset of sweating was 7.4 ± 1.6 min (range 4.8–10.3 min) after the start of cycling. Mass loss over the trial was 0.52 ± 0.07 kg with 206 ± 6 min between weighing.

#### 3.1.2. Skin Temperature

**Figure 4:**
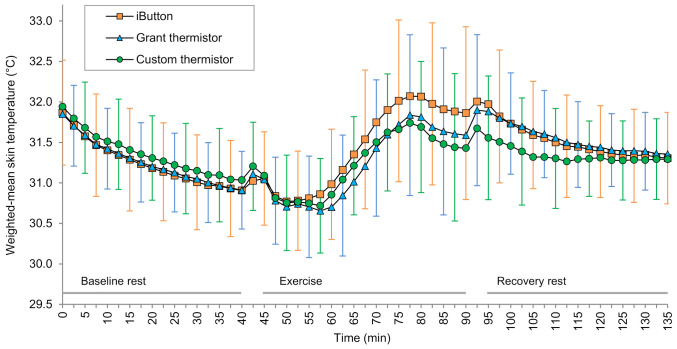
Weighted-mean skin temperature throughout baseline rest, cycling exercise, and recovery rest. Data are group mean (n = 14), and error bars indicate the standard deviation. For clarity of display, data are shown at intervals greater than the sampling frequency and the standard deviation for each sensor is shown at alternating time points; orange, blue, and green error bars correspond to iButton, Grant thermistor, and custom thermistor data, respectively.

**Figure 5:**
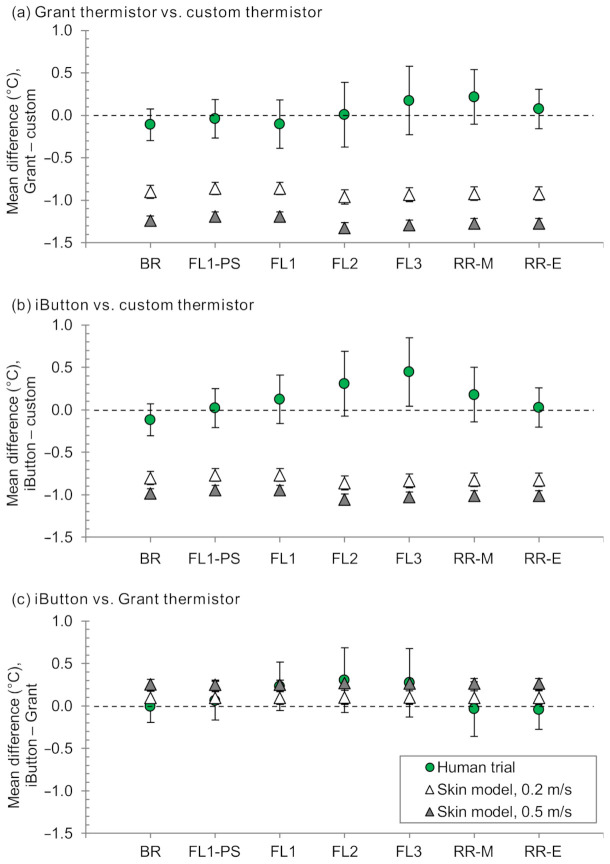
Mean differences between skin temperature sensors (**a**–**c**) during the human trial (weighted-mean skin temperature; n = 14) and skin model experiments (individual sensor temperature; n = 5 for each air velocity condition). Error bars indicate 95% confidence intervals. Negative differences indicate the first sensor listed being cooler than the second sensor listed. BR, end of baseline rest; FL1-PS, fixed-load cycling 1 pre-sweating; FL1–FL3, end of fixed-load cycling 1–3; RR-M, middle of recovery rest; RR-E, end of recovery rest.

No *T*_sk_ sensors were observed to have detached during the trials. One Grant thermistor had a technical issue, causing an offset during the baseline rest period of one experimental session. The data following resolution of the issue was used to estimate the offset and, therefore, estimate the baseline data for that sensor. All original sensors were used throughout the study, and datasets were otherwise complete. Weighted-mean *T*_sk_ from the three *T*_sk_ sensor types are shown in Figure 4, and the inter-sensor mean differences are shown in Figure 5. For the periods summarized, mean systematic differences between the three *T*_sk_ sensor types were always <0.3 °C during baseline and recovery rest and <0.5 °C during exercise. The largest mean difference was the iButton being 0.45 °C warmer than the custom thermistor at the end of exercise (Figure 5).

### 3.2. Thermal Skin Model

In one experimental run, the tape partially detached from the model surface, causing the wired *T*_sk_ sensor (Grant thermistor) to partially lift off the model surface; this experimental run was excluded, and a repeat run was performed. There were no other known technical issues with any sensors throughout the experimental runs, and datasets were, therefore, complete.

#### 3.2.1. Thermal Skin Model Comparison with the Human Trial Data

Mean differences among the three *T*_sk_ sensors are presented in Figure 5, comparing the model-derived values to those from the human trial. Model-derived differences were consistently greater in magnitude than the human trial for comparisons involving custom thermistors (Figure 5a,b). For the comparison of iButtons and Grant thermistors, model-derived mean differences were always within 0.4 °C of the corresponding mean differences from the human trial (Figure 5c). Indeed, for these two *T*_sk_ sensors, the model-derived differences with 0.2 m·s^−1^ air velocity were particularly close to the human trial for baseline rest, pre-sweating fixed-load cycling, and recovery rest (0.0–0.1 °C for skin model 0.2 m·s^−1^ vs. human trial), and the model-derived differences with 0.5 m·s^−1^ air velocity were particularly close for fixed-load cycling after the onset of sweating (<0.1 °C for skin model 0.5 m·s^−1^ vs. human trial).

#### 3.2.2. Temperature Disturbance

**Figure 6:**
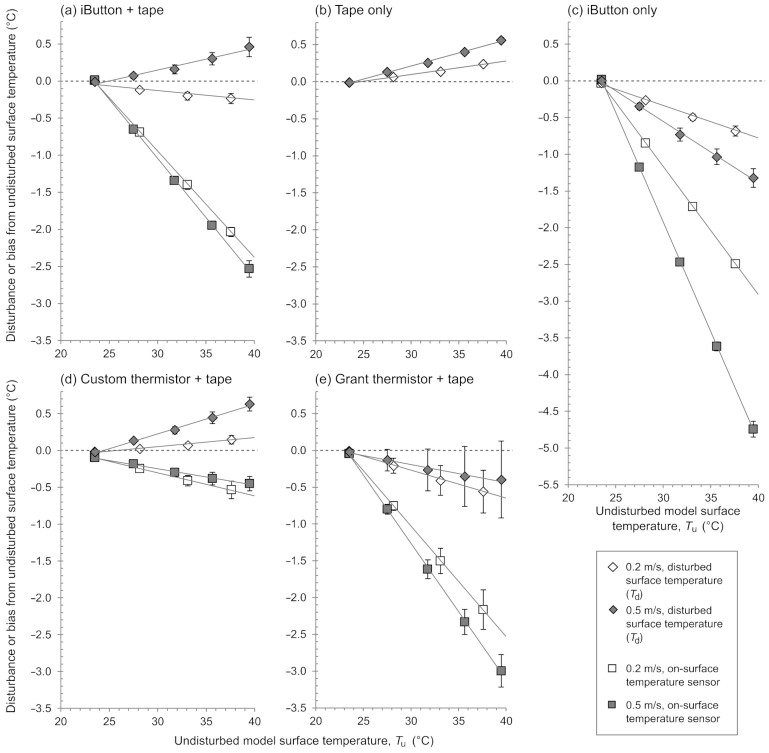
Magnitude of the local temperature disturbance caused by the surface skin temperature sensor and/or attachment and magnitude of the bias of the surface skin temperature sensor, each versus the model undisturbed temperature, under each air velocity condition (0.2 and 0.5 m·s^−1^) and for each surface condition (**a**–**e**). Plotted data points and error bars are mean ±95% confidence intervals (in some cases, the confidence intervals are too small to be visible in the figure); solid gray lines are regression lines fitted to mean data for illustrative purposes. Data beyond 40 °C (the last steady state for 0.2 m·s^−1^ air velocity) are not shown here to maintain emphasis on a physiological temperature range. *T*_d_, disturbed local surface temperature of the model; *T*_u_, undisturbed local surface temperature of the model.

When the surface of the bare skin model was warmer than the environmental air, the presence of a *T*_sk_ sensor and/or attachment caused a local temperature disturbance that increased in magnitude in proportion to the difference between the surface and environmental air temperatures (Figure 6). Coverage with tape only or with the custom thermistor and tape caused the underlying surface (i.e., *T*_d_) to be warmer than a bare surface under otherwise equivalent conditions (i.e., *T*_u_; Figure 6b,d), whereas the iButton without tape and the Grant thermistor and tape caused *T*_d_ to be cooler than *T*_u_ (Figure 6c,e). For the iButton and tape, the *T*_d_ was warmer than the *T*_u_ with an air velocity of 0.5 m·s^−1^ but lower than *T*_u_ with an air velocity of 0.2 m·s^−1^ (Figure 6a). The iButton only with an air velocity of 0.5 m·s^−1^, caused the greatest disturbance (e.g., −0.6 °C at an equivalent to *T*_u_ of 31 °C), while the custom thermistor and tape and tape only each at an air velocity of 0.2 m·s^−1^, caused the least disturbance (e.g., both +0.1 °C at an equivalent to *T*_u_ of 31 °C; Figure 6).

#### 3.2.3. Measurement Bias

When the surface of the skin model was warmer than the environmental air, the temperatures measured by the *T*_sk_ sensors were consistently lower than both the *T*_u_ and *T*_d_. The magnitude of the bias increased in proportion to the difference between the model surface and environmental air temperatures (Figure 6). To gauge the relevance these biases at a realistic *T*_sk_ reading, estimates are given in Table 1 to indicate the bias expected at a fixed *T*_sk_ sensor temperature of 31 °C. The iButton without tape showed the greatest bias from both *T*_d_ and *T*_u_ with, for example, these un-taped iButtons measuring 31 °C when the underlying *T*_d_ was 33.3 °C and the corresponding *T*_u_ was 34.2 °C (each at 0.5 m·s^−1^ air velocity). The custom thermistor and tape showed the least bias from both *T*_d_ and *T*_u_ with, for example, these custom thermistors measuring 31 °C when the underlying *T*_d_ was 31.4 °C and the corresponding *T*_u_ was 31.3 °C (each at 0.2 m·s^−1^ air velocity).

## 4. Discussion

Given the impracticality of using human skin in vivo for frequent or detailed assessments or comparisons of *T*_sk_ sensors, we sought to develop, evaluate, and utilize a synthetic skin model with realistic thermal properties as a benchtop alternative. There are two major outcomes from this work: First, the thermal skin model is promising for practical inter-sensor comparisons of common *T*_sk_ sensors (Grant thermistors and iButtons), although further work is required to understand why inter-sensor comparisons involving the custom *T*_sk_ sensors were dissimilar for the skin model versus human trial. Second, the skin model can be used to gain insight into errors associated with *T*_sk_ measurement. In particular, this skin model facilitated the manifestation of local surface temperature disturbances when using *T*_sk_ sensors, and results indicated that measurements from those *T*_sk_ sensors typically differ from both the underlying disturbed surface temperature and from the temperature of that underlying surface in its undisturbed state (i.e., when not being measured by the *T*_sk_ sensor). Demonstrating and characterizing local temperature disturbances alongside measurement errors with a benchtop tool provides a practical platform from which to explore the magnitude of such effects for a variety of *T*_sk_ sensor types and, where relevant, explore error mitigation methods (e.g., corrections or new sensor design).

### 4.1. Evaluation of Thermal Skin Model by Comparison with Human Trial

Here we have assumed having no practical ‘gold standard’ human *T*_sk_ measurement method. Accordingly, the skin model was evaluated by comparing the magnitude of mean inter-sensor differences determined on the skin model with the magnitude of those same inter-sensor differences determined on human skin during rest and cycling exercise. The skin model and the human trial were similar (within 0.4 °C) for mean inter-sensor differences between the Grant thermistors and iButtons, although this was not so for the comparisons including the custom thermistor.

It should be noted first that inter-sensor differences in the human trial were calculated using a four-site (four-sensor) weighted-mean *T*_sk_, whereas the inter-sensor differences from the skin model experiments were calculated using single-site (single-sensor) measurements. This approach was considered reasonable here, because the tighter control of experimental conditions afforded in the skin model experiments meant that temperature variability was considerably lower; in the human trial, the greater site-to-site, moment-to-moment variability is mitigated in part by averaging over the four sensors at the four body sites. Further, using weighted-mean *T*_sk_ is typical of human trials in the published literature and is typical in the assessment of thermal strain [3]. 

The similarity of the mean inter-sensor differences for the comparison between Grant thermistors and iButtons is an encouraging step in developing skin models that serve as practical, low-cost alternatives to human trials. An interesting observation was the closeness of the model-derived differences with 0.2 m·s^−1^ air velocity when compared to the human trial during periods where thermal sweating was not present (baseline rest, before the onset of sweating during fixed-load cycling, and from midway through recovery rest) and the closeness of the model-derived differences with 0.5 m·s^−1^ air velocity when compared to the human trial during periods where thermal sweating was present (fixed-load cycling after the onset of sweating; Figure 5). Indeed, considering only these combinations, 0.1 °C was the maximum difference (skin model vs. human trial) for mean differences between the Grant thermistors and iButtons.

The porous medical tape used throughout this work could be wetted during thermal sweating in the human trial, likely resulting in greater heat loss from the sensor via evaporative cooling [28,41]. Considering non-evaporative heat transfer when the air temperature is lower than the skin or surface temperature, a greater air velocity will result in greater heat loss from the *T*_sk_ sensor. While not implemented for this purpose, it may be that the greater of the two air velocity conditions within the skin model experiments resulted in additional heat loss analogous to that associated with evaporative cooling in the human trial. If, indeed, the case for the conditions used in the present study, this observation is unlikely to be generalizable but does indicate that test conditions for the future use of skin models should encompass variations that mimic dry and wet surface conditions if dry and wet surface conditions are expected during actual end use. Key simplifications of the skin model used in this work included that the surface of the model was in a fixed horizontal position and perpendicular to forced convection, and there was no attempt to simulate sweating. Future work could be observational, such as investigating effects of model orientation and the presence of surface moisture that simulates sweating, or analytical by modeling various modes of heat and/or evaporative transfers, sensor sizes, underlying tissues, sensor materials, and transient conditions in addition to steady states.

The custom thermistor had the greatest accuracy (least bias versus *T*_u_) of the three *T*_sk_ sensor types used here based solely on the outcome of the skin model experiments (Section 4.2), although this could not be translated to in vivo *T*_sk_ measurements when considering the magnitude of relative differences: the mean differences of the custom thermistor versus each Grant thermistors and iButtons were larger using the skin model than when using human participants (Figure 5). Therefore, when an inter-sensor comparison involved the custom thermistor, it is possible that the differences were overestimated in the case of the skin model, the differences were mitigated in the case of the human trial, or a combination thereof. 

A feature that could contribute to overestimation of differences (vs. custom thermistors) when using the skin model is that the same silicone elastomer was used for both the skin model and to encapsulate the custom thermistors (*T*_sk_ sensors), based on this material having thermal properties that reasonably approximates human skin [32,42]. The method of production meant that the top surface of the skin model and the bottom surface of the custom thermistor were each flat and smooth, and, in combination with being the same material, the thermal contact resistance may have been disproportionally low for the custom thermistor on the skin model compared to the custom thermistors on the human skin or compared to the Grant thermistors or iButtons on the skin model. Indeed, a practical observation during the experiments was that using the custom thermistors on the skin model created a kind of ‘seal’ at the model-senor interface.

In contrast, a feature that could contribute to mitigation of the inter-sensor differences (vs. custom thermistors) for human skin in vivo is the flexibility of the custom thermistors. The Grant thermistors and iButtons were each rigid (comprising stainless steel), and it was observed during the human trial that the iButtons and Grant thermistors appeared to depress into the skin more than the custom thermistors. Depression of a temperature sensor into the skin is associated with an otherwise higher measured temperature [43,44], which is consistent with the Grant thermistors and iButtons being closer to the custom thermistors than would be estimated based on the skin model. Another possibility is the influence of moisture buildup under the sensor acting to decrease thermal contact resistance in the case of human skin versus ‘dry’ skin models. The use of an interfacial medium to simulate the buildup of moisture is recommended when using *T*_sk_ sensors with a metallic contact surface in combination with a physical model also with a metallic surface [22]. An interfacial medium was not used in the present study for a number of reasons, including that the skin simulant used here was not hard or incompressible like the metallic models used elsewhere [21,22,29,30,31] and so that the results presented here can act as a baseline, following which modification of other model variables can be investigated and compared. Indeed, the choice of interfacial medium may itself also influence the outcome. A skin model that allows surface wetting to simulate sweating or transepidermal water loss should obviate the need to consider other interfacial mediums.

It was encouraging that the mean inter-sensor differences among the three *T*_sk_ sensors remained within ±0.5 °C in the human trial. That said, the measurement context of these relatively small differences was that the sensors were affixed to the participant using the same type of tape attachment, by the same experimenter in a systematized fashion, and with randomized relative placement of the sensors within each body site. Furthermore, all sensors were calibrated prior to use using the same instruments and processes. While this approach is expected within a method comparison study, such small differences cannot necessarily be expected when these mitigating factors are not also present, which is possible across different studies or measurement applications. Among 172 studies (published 2011–2016) involving physical activity, sport, or exercise and in which *T*_sk_ data were reported, the method of attachment was not reported or not clear in over half of those studies [10]. For over 90% of these studies, it was unclear whether the *T*_sk_ sensors had been calibrated. 

### 4.2. Thermal Skin Model Use for Understanding Local Temperature Disturbance and Measurement Error

The *T*_sk_ sensors investigated here exhibited linearly increasing bias concomitant with increasing surface-to-environment temperature gradient, a pattern consistent with previous work using aluminum models [21,22]. By using a skin model with thermal properties that better approximate human skin, the findings here expand on previous work by showing that using *T*_sk_ sensors caused a local temperature disturbance and by delineating two distinct forms of bias: bias from *T*_u_ and bias from *T*_d_ (Figure 1).

Our contention is that *T*_u_ represents the best estimate of the ‘true’ surface temperature, and thus, it is bias from *T*_u_ that represents the best estimate of *T*_sk_ sensor systematic error during use. Bias from *T*_d_ is perhaps only important in that *T*_sk_ sensor systematic error (i.e., bias from *T*_u_), itself, manifests from bias from *T*_d_ in combination with magnitude of the local temperature disturbance. The challenge here is that, in practice (outside the confines of these model experiments), it is only bias from *T*_d_ that is directly paired spatially and temporally with the *T*_sk_ sensor measurement, while the magnitude of the local temperature disturbance and the associated bias from *T*_u_ are somewhat hypothetical entities.

As indicated above, this characteristic of the current silicone elastomer model to facilitate local temperature disturbances contrasts metallic models in which local temperature effects are expected to be negligible due to the thermal conductivity being a different order of magnitude (e.g., aluminum ~230 W/(m∙K) versus human skin ~0.2 to 1.0 W/(m∙K)). One other experimental study had been located in which both the temperature bias and local modification were measured, although the surface sensor *T*_sk_ used in that experiment was designed for intermittent measurements, being manually held in place rather than being affixed in some way [45].

The thermal conductivity of the skin model used in this work (~0.23 W/(m∙K)) was within the range of values reported for human skin, although at the lower end of this range [42]. Skin perfusion increases with increasing *T*_sk_; hence, the effective thermal conductivity of the skin can approach or exceed 1 W/(m∙K) when warm and highly perfused [42]. Accordingly, additives could be used to tailor the thermal properties of the current skin model to investigate if changing the thermal conductivity within the range of ~0.2 to 1.0 W/(m∙K) influences the findings regarding local temperature disturbances and measurement biases. That said, previous numerical modeling indicated that, for an insulated *T*_sk_ sensor, the steady state skin surface temperature profiles were only negligibly influenced by perfusion rate [18].

In the present study, all sensors were attached using the same clinical tape, and the sensors were not vastly dissimilar in terms of physical size and shape. It would be useful for future work to include more diversity in the sensor setups being compared, from sensor systems smaller than those used here (e.g., [46]) through to sensors integrated into bulkier wearable devices such as ‘smart’ watches, bands, or patches [47]. From a practical perspective, this work justifies the ongoing development of sensor systems with the explicit intention of mitigating errors such as those described here. Reducing the size of the sensors is one way to mitigate errors, although such sensors must remain robust enough for practical use and need to balance reductions in size with susceptibility to point-to-point temperature variations within a local skin site. It is reasonable to expect that greater physical differences between sensor systems may lead to larger inter-sensor differences between those sensor systems, particularly during thermal sweating, so temperature data from larger devices must be treated with caution unless validity has been otherwise demonstrated. 

Furthermore, only temperature steady states were considered in the present study; future work could include a time component with rates of temperature change that are physiologically relevant. Environmental temperatures other than the temperate conditions used here (~24 °C) are equally important. That said, the linear relationships here between observed errors and the temperature difference between model surface and the environmental temperature are consistent with temperature errors in previous works [21,22], indicating that, all else equal, this temperature difference is, itself, more important than the absolute environmental temperature.

### 4.3. Toward Universal Comparability and Long-Term Sustainability of Datasets

In the present study, we have identified some idiosyncrasies of measurement system relevant for the quantification of *T*_sk_. More work is required to confirm that the local temperature disturbances and biases identified here are, indeed, representative of in vivo measurements; however, this work more widely illustrates that ongoing attention is required regarding the accurate acquisition of data in parallel with the end application of those data. Here, we have intentionally avoided specifying limits of practical relevance for measurement error, because the scope of this work was focused on understanding measurement errors independent of data end use. Future discussion is required around reasonable limits for acceptable error for given data end uses.

With contemporary interest in wearable technology, the increasing availability of large and personalized datasets, and the expanding potential of modeling physiological data for applications such as real-time feedback or predictive tools, the quality of the underlying data is important for establishing the quality of the use of that data. Efforts to understand and minimize errors or inconsistencies in measurement will be valuable, in that data models developed will need to be resistant to the propagation of errors, whereby small errors can become larger or more influential. Similarly, when considering the long-term sustainability of specific inferences, data models, or algorithms, it is clear that the outcome needs to be resistant to or independent of the specific measurement system used (e.g., types of temperature sensors and attachments) *and* to the effects of changing parts of a given measurement system with time (e.g., iterations of sensor or device design, material selection, changing commercial availability of parts). Independence of the measured value from the specific sensor system used also facilitates transfer of data or inferences to other parties. 

One way forward is continuing to improve the measurement methods, whereby increasing the accuracy of sensors should lead to convergence of those sensor systems near the ‘true’ value for any given measurement context, or to the selection of specific sensor types that are shown to have superior validity. Another approach is having a standardized tool and method for baseline surface temperature measurements that allow intra- and inter-sensor measurements to be documented, reported, and tracked or compared over time. In principle, this establishment of baseline comparability should be encompassed via sensor calibration. However, the work here and elsewhere [22] illustrates that contact *T*_sk_ sensors calibrated by conventional means—that is, in a uniform thermal environment (e.g., water bath)—are still prone to errors when there is a temperature gradient across the measurement system, as is typically the case during measurement of human skin. A benchtop skin model is one candidate for this latter approach and can serve as a highly controlled medium for baseline *surface* comparisons that supplement conventional sensor calibration information and can supplement comparisons done in vivo [48].

Using human skin for assessing *T*_sk_ sensors increases the ecological validity of the findings but also has a number of practical and logistical constraints. Beyond resource and ethical considerations, one pertinent constraint is that comparisons of different sensors cannot be done both at the exact same site *and* time. This constraint also applies to other approaches including skin models; however, other physical and physiological factors complicate the situation in the case of measurements made in vivo. These factors include spatial heterogeneity of the underling tissues (e.g., dermis, epidermis, hypodermis, skeletal muscle, bone) or structures (blood vessels, sweat glands), effects of the orientation of the sensor (e.g., exposure to different local air movements), and interaction with sweat and associated evaporative cooling. This complexity of in vivo measurements, combined with the difficulty in determining what the ‘true’ reference temperature should be, supports the development of suitable skin models for practical intra- and inter-sensor comparisons and for understanding the impact of *T*_sk_ sensors on the skin itself during the measurement process.

## 5. Conclusions

The thermal skin model was suitable for replicating the in vivo inter-sensor mean differences for the comparison of two common *T*_sk_ sensors (iButtons and Grant thermistors; within 0.4 °C for in vivo vs. skin model) but not for those involving a third *T*_sk_ sensor (custom thermistors). In speculation, this finding for comparisons involving the custom thermistors could be an anomaly due to the same material being used to make the skin model and to encapsulate the custom thermistors. Results from the skin model also demonstrate that using contact *T*_sk_ sensors can cause a local temperature disturbance and that those *T*_sk_ sensors become increasingly biased concomitant with an increasing surface-to-environment temperature gradient. Following the identification of two forms of *T*_sk_ sensor bias (bias from *T*_d_ and bias from *T*_u_), we contend that bias from *T*_u_ represents the best estimate of *T*_sk_ sensor systematic error during use, and accordingly, this form of bias is important for future sensor characterization, design, or correction. Accounting for evaporative heat transfers, changes in contact thermal resistance (i.e., buildup of moisture between skin and sensor) and further validation of model insights versus human skin are logical next steps for future work.

## Figures and Tables

**Table 1:** Calculated estimates of bias (mean (95% confidence intervals)) for a fixed surface skin temperature sensor of 31 °C, expressed as difference from the disturbed reference temperature (*T*_d_) and from the corresponding undisturbed reference temperature (*T*_u_). Negative values for bias indicate the skin temperature sensor is cooler than the corresponding reference temperature.

	Custom Thermistor + Tape, °C	Grant Thermistor + Tape, °C	iButton + Tape, °C	iButton Only, °C
0.2 m·s^−1^ air velocity				
Bias, from *T*_d_	−0.4 (−0.5 to −0.3)	−1.0 (−1.3 to −0.8)	−1.1 (−1.1 to −1.1)	−1.2 (−1.2 to −1.2)
Bias, from *T*_u_	−0.3 (−0.4 to −0.3)	−1.4 (−1.6 to −1.2)	−1.3 (−1.3 to −1.2)	−1.6 (−1.7 to −1.6)
0.5 m·s^−1^ air velocity				
Bias, from *T*_d_	−0.6 (−0.6 to −0.5)	−1.5 (−2.0 to −1.1)	−1.6 (−1.7 to −1.6)	−2.3 (−2.4 to −2.1)
Bias, from *T*_u_	−0.3 (−0.3 to −0.2)	−1.8 (−1.9 to −1.6)	−1.4 (−1.5 to −1.4)	−3.2 (−3.3 to −3.1)

**Figure A1:**
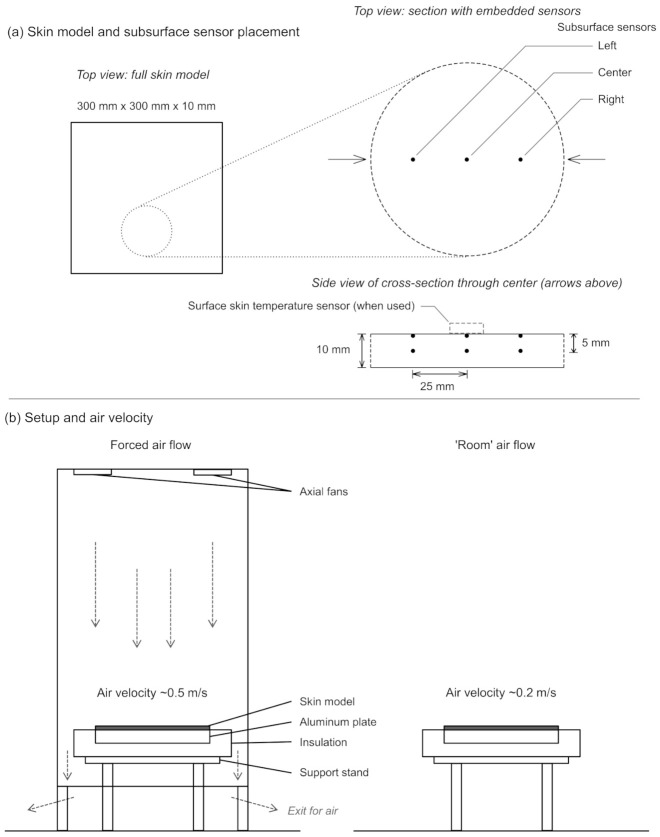
Setup of the silicone elastomer thermal skin model (**a**) and the overall setup for controlling the temperature (via water-perfused aluminum plate) and air velocity (fans or no fans (**b**)). The reference temperature used during this experiment was from the center of the three directly subsurface temperature sensors embedded in the skin simulant (**a**; see description in Appendix A.2, below). When a surface skin temperature sensor and/or attachment was used, it was positioned directly above the middle subsurface temperature sensor. The second row of sensors within the silicone elastomer skin (5 mm beneath the surface) enabled the estimation of temperature gradient through the model.

**Figure A2:**
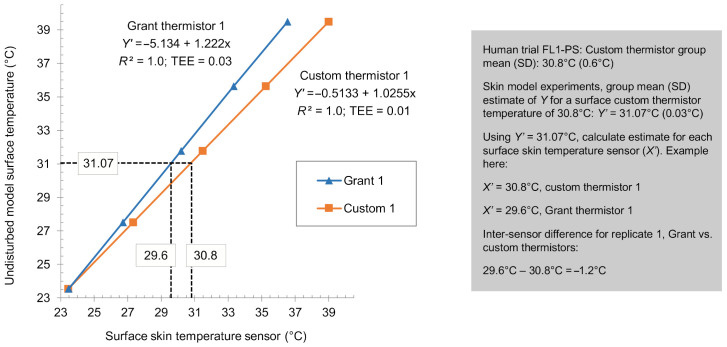
Estimation of required model surface temperature and resultant temperatures of the skin temperature sensors in an example inter-sensor comparison (Grant thermistor vs. custom thermistor). Plotted data are the first replicates for each respective skin temperature sensor in the skin model experiments under the air velocity condition of 0.5 m·s^−1^. See the main text (Section 2.5.3) for more information. FL1-PS, fixed-load cycling 1 pre-sweating; SD, standard deviation; *R*^2^, coefficient of determination; TEE, typical error of the estimate.

## Data Availability

The data that support the findings of this study are available upon reasonable request.

## Appendix A. Further Detail about the Thermal Skin Model

### Appendix A.2. Establishing the Undisturbed Reference Temperature

The thermal skin model was made with a row of three temperature sensors embedded directly beneath the surface of the skin model (Figure A1), because it was initially expected that the lateral sensors would be used as the *T*_u_ reference for the runs when the center sensor was, itself, covered by the surface *T*_sk_ sensor and/or attachment (i.e., center sensor becoming *T*_d_). In order to designate the sensor(s) to be used as the *T*_u_ reference, data from the five experimental runs with no covering (i.e., bare skin model) were used to characterize the similarity between and within the three directly subsurface temperature sensors at each temperature steady state. The summary data from the *uncovered runs*, only, are given in Table A1. Considering the left and right subsurface sensors, the left was more consistent with the center subsurface sensor: mean differences across all steady states for this comparison were −0.01 to −0.19 °C for 0.2 m·s^−1^ air velocity and <0.01 to 0.02 °C for 0.5 m·s^−1^. However, the center subsurface sensor showed very little inter-run, within-sensor variation at each steady state, with the biggest absolute difference within any steady state being 0.05 °C; all SD were ≤0.02 °C. That is, the experimental setup and temperature steady states were found to be very consistent, with excellent run-to-run repeatability. Because these inter-run, within-sensor differences for the center reference sensor were so small, the mean values for the center subsurface steady states during these uncovered runs were used as the *T*_u_ reference for all covered experimental runs. In this way, the *T*_d_ and *T*_u_ came from the identical sensor and position, albeit from separate experimental runs.

**Table A1:** Steady state mean temperatures (°C) for the directly subsurface temperature sensors during the uncovered experimental runs (bare model surface) under each air velocity condition (0.2 or 0.5 m·s^−1^). Data are mean (SD) for n = 5 replicates.

	0.2 m·s^−1^ Condition			0.5 m·s^−1^ Condition		
Steady state	Subsurface sensor position			Subsurface sensor position		
	Center	Left	Right	Center	Left	Right
1	23.50 (0.01)	23.49 (0.01)	23.51 (0.01)	23.54 (0.02)	23.52 (0.02)	23.55 (0.02)
2	28.21 (0.01)	28.16 (0.01)	28.16 (0.01)	27.59 (0.02)	27.59 (0.02)	27.48 (0.02)
3	33.21 (0.01)	33.11 (0.02)	33.08 (0.01)	31.93 (0.02)	31.93 (0.02)	31.67 (0.04)
4	37.76 (0.01)	37.62 (0.02)	37.55 (0.02)	35.89 (0.02)	35.89 (0.02)	35.47 (0.03)
5	42.26 (0.02)	42.07 (0.02)	41.93 (0.02)	39.80 (0.02)	39.79 (0.02)	39.21 (0.04)

## Appendix B. Calibration of Temperature Sensors

All datasets from the calibration procedures were complete. Sensor-specific linear equations were satisfactory for correcting the raw temperature data, as indicated by a low typical error of the estimate (TEE; mean residual error from the fitted regression line) for each set of steady state vales.

- For the calibration of the *thermistors embedded in the skin simulant* (n = 6), the TEE was 0.002–0.004 °C.
- For the calibration of the *T**sk sensors prior to the skin model experiments* (n = 5 for each sensor type), the TEE was: custom thermistors, 0.03–0.04 °C; Grant thermistors, 0.01–0.02 °C; and iButtons, 0.02–0.05 °C.
- For the calibration of the *T**sk sensors prior to the human trial* (n = 4 for each sensor type), the TEE was: custom thermistors, all <0.01 °C; Grant thermistors, all <0.01 °C; and iButtons, 0.01–0.02 °C.

## Appendix C. Estimating Thermal Skin Model Inter-Sensor Differences

An example is given in Figure A2 illustrating:

- the estimation of the model surface temperature required to be approximately equivalent to a given period mean skin temperature from the human trial (in this example, the period immediately preceding the onset of sweating during fixed-load exercise), and
- the estimation of temperature for each respective surface *T*_sk_ sensor (in this example, a Grant thermistor and custom thermistor), which is then used to calculate that particular inter-sensor difference.

For each *T*_sk_ sensor replicate under each skin model air velocity condition, this process was repeated at each model surface temperature that corresponded to a period mean skin temperature from the human trial.
